# Genetic diversity and drug resistance surveillance of *Plasmodium falciparum* for malaria elimination: is there an ideal tool for resource-limited sub-Saharan Africa?

**DOI:** 10.1186/s12936-019-2844-5

**Published:** 2019-06-26

**Authors:** Tobias O. Apinjoh, Amed Ouattara, Vincent P. K. Titanji, Abdoulaye Djimde, Alfred Amambua-Ngwa

**Affiliations:** 1 Department of Biochemistry and Molecular Biology, University of Buea, Buea, Cameroon; 2 School of Medicine, University of Maryland, College Park, Baltimore, USA; 3 Faculty of Science, Engineering and Technology, Cameroon Christian University, Bali, Cameroon; 4 Malaria Research and Training Centre, University of Science, Techniques and Technologies of Bamako, Bamako, Mali; 5 Medical Research Council Unit The Gambia at LSHTM, Banjul, The Gambia

**Keywords:** Genetic diversity, Drug resistance, Monitoring, *Plasmodium falciparum*

## Abstract

The intensification of malaria control interventions has resulted in its global decline, but it remains a significant public health burden especially in sub-Saharan Africa (sSA). Knowledge on the parasite diversity, its transmission dynamics, mechanisms of adaptation to environmental and interventional pressures could help refine or develop new control and elimination strategies. Critical to this is the accurate assessment of the parasite’s genetic diversity and monitoring of genetic markers of anti-malarial resistance across all susceptible populations. Such wide molecular surveillance will require selected tools and approaches from a variety of ever evolving advancements in technology and the changing epidemiology of malaria. The choice of an effective approach for specific endemic settings remains challenging, particularly for countries in sSA with limited access to advanced technologies. This article examines the current strategies and tools for *Plasmodium falciparum* genetic diversity typing and resistance monitoring and proposes how the different tools could be employed in resource-poor settings. Advanced approaches enabling targeted deep sequencing is valued as a sensitive method for assessing drug resistance and parasite diversity but remains out of the reach of most laboratories in sSA due to the high cost of development and maintenance. It is, however, feasible to equip a limited number of laboratories as Centres of Excellence in Africa (CEA), which will receive and process samples from a network of peripheral laboratories in the continent. Cheaper, sensitive and portable real-time PCR methods can be used in peripheral laboratories to pre-screen and select samples for targeted deep sequence or genome wide analyses at these CEAs.

## Background

Malaria continues to cause significant morbidity and mortality across Africa, despite the intensification of control interventions [1]. A worldwide decline in its burden was reported in 2015, with rebound and age shift in morbidity as control and interventions are scaled up [2–6]. Malaria has historically resurged as interventions are withdrawn or perturbed by socio-political factors [7]. In fact, recent reports suggest that malaria incidence increased between 2014 and 2017 in several regions across sub-Saharan Africa (sSA), where most of the global investments in control and elimination is spent [1]. This is a cause for concern and an indication that further knowledge on the impact of interventions on parasite and vector populations, including parasite’s mechanisms of adaptation to environmental and interventional pressures are needed to refine control and elimination strategies.

The success of *Plasmodium falciparum* as a parasite has been attributed partly to its enormous genetic diversity [8–10], that allows it to adapt to anti-malarials [11] and hinder the development of an effective vaccine [12]. Parasite populations even respond to specific interventions, such as rapid diagnostic tests, human host immune pressure and mosquito vector environment [13–15]. The molecular mechanisms and markers of these phenomena need to be monitored. Identifying and monitoring markers of adaptive mechanisms against anti-malarials are of particular interest as anti-malarial chemotherapy and chemoprophylaxis have been key in reducing mortality and morbidity from malaria [16, 17].

Currently, malaria chemotherapy relies mainly on the sustained efficacy of artemisinin-based combination therapy (ACT), which are the recommended drugs for the treatment of adults and children with uncomplicated *P. falciparum* infection [18]. ACT combines artemisinin derivatives and other long-acting anti-malarials, with a different mechanism of action, to maximize the chances of eliminating parasites during treatment, but also to limit the possibility of a spontaneous mutation that will confer resistance to both drugs [19, 20]. Resistance to artemisinin derivatives (ART-R) already emerged and led to reduced parasite susceptibility to artemisinin derivatives [21] that manifests as increased parasite clearance half-life [22] or persistence of microscopically detectable parasites on day 3 post-ACT treatment [19]. Furthermore, ACT resistant *P. falciparum*, with reduced susceptibility to both artemisinin derivatives and partner drugs, is now a public health concern in South East Asia (SEA) [23, 24]. This highlights the parasite’s ability to adapt to and successfully evade multiple drug interventions, particularly in SEA. Though the delayed clearance phenotype of artemisinin resistance has not been widely observed in Africa, continuous surveillance for drug efficacy and molecular markers of resistance provide one of the sensitive approaches for early detection of any de novo emergence or importation into sSA [25].

Molecular surveillance can help in determining the impact of interventions on patterns of genomic variation, population structure and gene flow between *P. falciparum* from similar or distinct geographical, ecological and interventional settings in Africa [26–28]. Molecular epidemiology studies also have several implications. Firstly, they serve to improve understanding of the parasite’s biology and new functional genetic variants can be characterized within and between *P. falciparum* populations [29–31]. Secondly, they can permit the identification of novel loci associated with clinically relevant phenotypes such as resistance to new and candidate drugs [32, 33]. This was recently exemplified following the emergence of delayed *P. falciparum* clearance for artemisinin treatment, where mutations were identified in *kelch13* propeller gene (*Pfk13*) [34] and shown to be spreading across SEA [35]. Subsequently, molecular epidemiology of variants of *kelch13* were employed to time the emergence and evolution of the phenotype across SEA [36, 37]. Regular monitoring of these markers across malaria endemic regions will ensure its containment and prevent its emergence or spread to Africa, as was the case with previous anti-malarials [38–40]. Beyond the parasite, molecular surveillance can be extended to vectors for development of tools to monitor and evaluate interventions against the vector, particularly changes in species composition, structure and the emergence and spread of insecticide resistance [41].

This review aims to examine the current tools for monitoring the emergence of anti-malarial drug resistance and the assessment of *P. falciparum* diversity for malaria elimination in sSA. A two-tier approach for widening the application of molecular epidemiology into intervention approaches is presented; the deployment of the polymerase chain reaction (PCR) and quantitative PCR (qPCR) in peripheral laboratories and the application of targeted or whole genome sequencing as a sensitive genetic epidemiology tool in specialized research centres or Centres of Excellence in Africa (CEA). This can be made possible with new portable PCR machines in the cost range USD5000–10,000, which falls within The World Academy of Science (TWAS) and International Foundation of Science (IFS) grants widely accessible to sSA scientists.

## Methods

Original peer-reviewed publications were accessed via PubMed (www.pubmed.gov) and Google Scholar (www.google.scholar.com) using the keywords ‘*Plasmodium falciparum* and “genetic diversity” or “anti-malarial drug resistance”. In the following sections the authors present their findings and suggestions. The variety of approaches for drug resistance and diversity monitoring is presented in Table 1.

**Table 1:** Tools for drug resistance assessment and parasite diversity monitoring

Tools	Capacity	Resources required	Advantages	Disadvantages
Drug resistance				
Ex vivo/in vitro tests	Few samples	BSL2 for parasite culture, refrigerator, freezer, microscope, incubator	Several tests possible on same sample, no heavy equipment	Labor-intensive, expensive, generalization difficult due to different tests/methods, requires advance culture infrastructure
RSA/PSA				
TES	Hundreds	Refrigerator, freezer, microscope, incubator, PCR machine, gel electrophoresis, transilluminator	No heavy equipment required	Expensive
Molecular markers	Hundreds	PCR machine, Restriction enzymes, refrigerator, freezer,	Ease of sampling, storage and transport	Markers not always linked to clinical outcome, require good infrastructure and trained personnel
Parasite diversity monitoring				
msp/glurp typing	Hundreds	PCR machine, Gel electrophoresis, transilluminator	Simple to implement	Labour-intensive, subjective interpretation
DNA barcodes	Thousands	Real time PCR	Sensitive, robust	Requires relatively sophisticated TaqMan or HRM assay
Microsatellite typing	Thousands	Real time PCR	Very informative from large range of alleles per locus	Requires relatively sophisticated PCR. Lack of standardization in scoring alleles
TDS	Hundreds	Sequencer, Server capacity for data storage	Less subject to inherent bias	High cost, requires large data storage capacity, requires skilled personnel. Target ascertainment bias
Genome wide analysis	Hundreds	Sequencers, Server capacity for data storage	Access to whole genome variants including single nucleotide and structural polymorphisms	As for TDS but need for target DNA enrichment against human DNA, difficulty in constructing haplotypes, and low sensitivity to detect minority clones

*BSL2* biosafety level 2, *RSA* ring stage assay, *TES* therapeutic efficacy studies, *TDS* targeted deep sequencing

### Drug resistance monitoring

To pre-empt the emergence or spread of resistance to anti-malarial drugs in Africa, the genotype and phenotype of parasites from all malaria endemic populations need to be regularly monitored. Accurate molecular surveillance in elimination settings require sensitive diagnostics of low grade parasitaemia and unbiased sampling strategies that enables resolution of temporal and geospatial patterns. Thus, the detection of parasitaemia by histidine rich protein (HRP)-based rapid diagnostic test (RDT), for instance, will be insensitive and potentially exclude *P. falciparum* parasites lacking HRP2 and HRP3 genes [42]. Given available resources, three common approaches can provide samples; (a) community-based cross-sectional surveys designed to capture the demography and spatial specifications of the target population, (b) clinical surveys, in which samples from patients suspected or diagnosed of malaria at health facilities are collected and (c) therapeutic efficacy studies (TES) engaged by research groups or the National Malaria Control Programme (NMCP). Each of these sampling approaches can be biased by being non-random and unrepresentative of the population. This can be ameliorated by increasing the density of spatial and temporal coverage or analysing all positive samples for populations where the prevalence of *P. falciparum* is low [43].

The choice of drug resistance surveillance assay or combination of phenotypic and genotypic analyses depends on resources and the primary goal of the study and NMCPs [44–47]. Therapeutic efficacy studies remain the gold standard for anti-malarial susceptibility testing, but needs significant logistic organization [48]. The health budget, infrastructure and trained human resources in most sub-Saharan African countries are insufficient to support routine and wide application of TES. Typically, most NMCPs in sSA are supported by World Health Organization (WHO) and/or the Global fund for in vivo TES in limited number of sites. These provide samples for PCR correction and global genetic surveillance coordinated by the WHO. In vitro*/*ex vivo testing and genotyping of all tested samples will enrich drug efficacy data. Nevertheless, a subset of samples collected either from TES or cross-sectional surveys could be analysed by ex vivo/in vitro assays because of logistical constrains.

### Ex vivo/in vitro tests for resistance

Samples from community or clinical surveys or from TES can be tested ex vivo or in vitro for susceptibility to different anti-malarial compounds. In vitro assays assess cultured parasites, meaning that only a subset of the parasites collected in the sample that are adapted to laboratory culture conditions are analysed, in contrast to ex vivo for which fresh samples are immediately tested. These drug susceptibility tests offer a convenient approach to determine sensitivity to anti-malarials, as they are based on direct contact between parasites and test drugs over a range of concentrations. In addition, several drugs (including those at experimental stages) can be tested against the same parasite isolate. A major limitation is the need for functional cell culture facilities and equipment for measuring parasite growth, such as microscope, spectrophotometer, fluorimeter or cytometer. Most tests determine the inhibitory concentration that kills half of the cultured parasites (IC50). IC50 assays have only recently been standardized by World Wide Antimalarial Resistance Network (WWARN) and are not suitable for fast acting artemisinin derivatives. Hence, the in vitro ring-stage survival assay (RSA) and piperaquine survival assay (PSA) were developed for testing ART-derivatives and piperaquine, respectively [45, 49, 50]. RSA together with Pfk13 molecular characterization is recommended in surveillance of ART-R [48]. These are highly technical assays targeting *P. falciparum* early ring-stages with a burst of high drug concentration in vitro. They are also yet to be developed for low grade parasitaemia often found in asymptomatic malaria parasite infection. Like IC50 assays, they are largely unstandardized and require specialized cell biology skills and techniques to distinguish parasite phenotypes such as dormancy. Although it is impossible to standardize in vitro assays, a data analytical tool (IVART) has been developed by WWARN to standardize the analyses process and facilitate future comparability of data across different studies [51]. The lack of drug resistant standards for current and candidate drugs, the substantial laboratory infrastructure and need for highly trained personnel in cell biology and data analysis limits the wide adoption of in vitro testing across Africa [44]. The future development of short culture-independent bench top tests, which target parasite metabolic markers from pathways of drug resistance, could allow for wider adoption.

### Genetic markers of anti-malarial drug resistance

Trends in anti-malarial drug efficacy and resistance can also be assessed using molecular markers (Table 2). These can be good predictors of treatment outcome patterns at a population level [52–54]. Molecular markers have several practical advantages over in vitro tests, including the possibility of studying many isolates within a short time with DNA from dried blood spots (DBS), which are easy to collect, transport and store. There exists a wide panel of molecular markers, mostly associated with widely used previous first line anti-malarial drugs, quinolines and sulfadoxine–pyrimethamine (SP) [44]. These previous first-line drugs are still used for intermittent preventive treatment in pregnancy (IPTp) and seasonal malaria chemoprevention (SMC), or against co-transmitted *Plasmodium vivax*. Hence, their resistance markers remain relevant, especially as some alleles modulate efficacy of ACT [55]. Most marker alleles can be typed using technological variants of various methods, including; dot blotting, restriction fragment length polymorphism (RFLP), PCR/qPCR, mini/microarrays, targeted/whole-genome sequencing. Sample pooling strategies, advances in PCR and DNA sequencing technologies promise to improve the scalability of genotyping resistance marker and wider population coverage [56]. Next Generation Sequencing (NGS) in particular can now be achieved with as low as $22 per Gigabyte (Gb) on Illumina HiSeq 3000/4000 while Third Generation Sequencing with Pacific BioSciences, the most widely used long-read platform costs $1000 per Gb [57].

Emerging nucleic acid sequencing technologies such as the portable nanopore devices also have the potential to revolutionize next-generation sequencing approaches towards field-based genotyping in resource-constrained regions in sSA. However, nanopore assays specific to malaria parasite diversity and drug resistance markers are unavailable or only recently been developed despite clear applicability of this technology in outbreaks such as Ebola and Lassa fever in Africa [58, 59]. Developing field deployable assays for markers of ACT component drugs (artemisinin derivative in combination with either lumefantrine, amodiaquine, mefloquine, quinolines or sulfadoxine–pyrimethamine) in Africa would be most useful.

**Table 2:** Mode of action, targets and resistance mechanisms of drugs for *P. falciparum* malaria treatment and control in sSA

Drugs	Mode of action	Molecular markers of resistance	Resistance mechanism
Artemether (AM)	Not well understood, oxidative damage to proteins and lipids and/or targeting the phosphatidylinositol-3-kinase (PfPI3K)	*Pfk13*, *Pfmdr*-*1*, *Pfmrp*-*1*	Not clearly understood; SNPs, CNVs
Artesunate (AS)			
Dihydroartemisinin (DHA)			
Pyrimethamine	Inhibits folic acid synthesis	*Pfdhfr*	SNPs
Sulphadoxine		*Pfdhps*	SNPs
Amodiaquine (AQ), lumefantrine (LM)	Inhibits haem detoxification	*Pfcrt*	SNPs
		*Pfmdr*-*1*	SNPs
Mefloquine (MQ)			CNVs
		*Pfcrt*, *plasmepsin 2/3*	SNPs, CNVs
Quinine (QN)		*Pfmdr*-*1*, *Pfcrt*, *Pfmrp*-*1*, *Pfnhe*-*1*	SNPs
Piperaquine (PPQ)	Inhibits haem detoxification, inhibits one or more steps in the haemoglobin degradation		
Clindamycin	Inhibits protein synthesis	*Pfmdt*, *PftetQ*	CNVs
Doxycycline			

Recommended ACTs for treatment of malaria have artemisinin or its derivative combined with one or more drugs and include: AL, AS–AQ, AS–MQ, AS–SP and DHA–PPQ. As of 2016, most African countries use AL and AS–AQ, with some adding DHA–PPQ for uncomplicated malaria, AS, AM and QN for severe malaria and SP for IPTp (1)

*CNV* copy number variation, *Pfmrp*-*1 P. falciparum* multi-resistance protein 1 gene, *Pfnhe*-*1 P. falciparum* Na^+^/H^+^ exchanger-1, *Pfmdt P. falciparum* metabolic drug transporter, *PftetQ P. falciparum* tet Q GTPase

Mutations in the propeller domain of the Pfk13 gene have been validated as *P. falciparum* ART-R markers in SEA [34, 54]. SNPs in four other genes, namely, ferredoxin (PF3D7_1318100, fd), apicoplast ribosomal protein S10 (PF3D7_1460900, arps10), multidrug resistance protein 2 (*mdr2*) and chloroquine resistance transporter (*crt*) are also thought to be the backbone loci on which ART-R associated *kelch13* mutations are most likely to arise [54]. Though key *Pfk13* ART-R associated mutations are absent in sSA [38], typing panels could include known and candidate drug resistance loci whose allele frequencies are changing in some parts of sSA following ACT implementation [29, 60]. Artemether–lumefantrine (AL), for instance, is thought to select for *Pfcrt* and *Pfmdr1* wild type variants following treatment. Other markers could include variants of *Pfap2mu* (encoding clathrin-associated AP2 adaptor protein, µ subunit) and *Pfubp1* (encoding ubiquitin carboxyl-terminal hydrolase 1) found more frequently in *P. falciparum* isolates post-ACT [61]. Indeed, candidate SNP variants in *Pfap2mu* should be included in SNP panels for continental surveillance during ACT and for validation as a marker for ACT tolerance in African parasite populations.

The choice of genotyping panels is complicated by the variety of recommended ACT medicines, heterogeneously available across Africa. This presents a threat of partner drug resistance across a broad spectrum of anti-malarials. Already, mutations in *Pfcrt* and *Pfmdr1* are being selected by the most common ACT, artemether–lumefantrine (AL) and artesunate–amodiaquine (AS–AQ) [62]. Polymorphisms in both genes are also associated with structurally similar partner drugs; mefloquine, amodiaquine, piperaquine [63]. Moreover, *Pfcrt* mutations as well as *plasmepsin 2/3* copy number variations have been shown to confer *P. falciparum* resistance to piperaquine [64, 65]. The presence of these in African populations would affect deployment and efficacy of piperaquine-based ACT [66]. Monitoring of partner drug resistance markers is, therefore, particularly key for parasite elimination. When such markers are detected in the circulating parasite populations, treatment and preventative chemotherapy policy could be revised to switch to other drug combinations. Recommended drug-based preventive malaria interventions in Africa include; intermittent preventive treatment in infants (IPTi) and pregnant women (IPTp) with SP in areas of moderate-to-high malaria transmission in sSA and amodiaquine in combination with SP for SMC in the Sahel [16, 67]. However, resistance to SP is widespread and marked by mutations in the *P. falciparum* dihydropteroate synthetase (*Pfdhps*) and dihydrofolate reductase (*Pfdhfr*) genes. Though chloroquine, amodiaquine and SP are no longer first-line malaria treatment, mutations in *Pfmdr1*, *Pfdhfr*, *Pfdhps*, *Pfcrt* and candidate loci for artemisinin and piperaquine resistance are needed to assess the impact of current drug interventions.

The design of surveillance panels should consider the complex relationships between individual mutations and haplotypes with sensitivity to specific anti-malarials. For instance, the *Pfcrt* 76T mutant is essential for chloroquine resistance but is selected as *Pfcrt* CVIET or *Pfcrt* SVMNT haplotypes that confer fitness against chloroquine pressure [68]. Similarly, SP resistance is linked to the *Pfdhfr* IRN triple and *Pfdhps* double GE haplotypes selected by SP interventions [69]. Medium SNP typing panels could include these linked loci, while small panels could combine markers that maximize information for key resistance loci of anti-malarial treatments or chemoprevention. For example, countries using AL, could employ a small panel consisting of N86Y, K76T and C580Y for *Pfmdr1*, *Pfcrt* and *Pfk13*, respectively. This can be expanded to include *Pfdhfr* K540E and A581G to cover selection by IPTi/IPTp and SMC in the Sahel. These panels will gain from assays such as RFLP and allelic discrimination real time PCR by high resolution melting (HRM), which do not rely on heavy and expensive equipment. These techniques can achieve higher coverage given the wide availability of restriction and amplification reagents including isothermal polymerases. They could be extended to detect copy number variants, such as those in *plasmepsin* genes associated with sensitivity to piperaquine. The small/medium panels would be used at peripheral labs with limited capabilities, but all the markers should be assessed in CEAs.

### Parasite diversity monitoring

Single nucleotide polymorphisms and structural genetic variants of *P. falciparum* can serve to fingerprint infecting parasites and inform the effectiveness of drug treatments or vector interventions. Four major strategies have mostly been explored, each with advantages and limitations for wide adoption across sSA. These include: *msp/glurp* typing, microsatellite analysis, molecular (DNA) barcodes, targeted deep sequencing and genome-wide variation analysis.

### *msp/glurp* typing

Viriyakosol et al. [70] were the first to deploy a tool that exploits length polymorphisms in merozoite surface protein (*msp*) and glutamine rich protein (*glurp*) genes to assess the identity or genotype of infecting parasites. Two different *msp* markers are often used, *msp1* and *2*. This technique is particularly useful in determining the complexity of infection (COI), a measure of the effectiveness of intervention programmes. *Msp/glurp* typing are widely used in anti-malarial drug efficacy trials to distinguishing recrudescent parasites from new infections [71, 72]. It has been one of the most widely adopted techniques because of the availability of PCR and DNA electrophoresis equipment which are now portable. Furthermore, its sensitivity and reliability are improved in specialized laboratories by incorporating capillary electrophoresis and more recently qPCR-HRM [71, 73].

A major limitation of *msp/glurp* typing is lack of standardization of scoring and reporting formats that can allow for the comparison of results across different endemic site laboratories. The *msp/glurp* genotyping protocol is also labour-intensive, depends on the sensitivity of PCR, which may not amplify low abundance variants or result in artefacts [74]. In addition, the sensitivity is low when using agarose gels and the interpretation can be subjective, especially in high transmission areas in Africa where polyclonal infections will lead to multiple bands. Furthermore, *msp/glurp* genes are under immune selection pressure, which can skew the frequency of some allelotypes [30]. This could affect the accuracy of population structure and transmission patterns inferred from these loci. Notwithstanding the above limitations of *msp/glurp* typing, it remains a popular method for fingerprinting across endemic regions in sSA. Its continuous use will benefit from allele size reference standards derived from culture adapted reference isolates from major *P. falciparum* populations across Africa.

### Microsatellite analysis

Microsatellites are tandem repeats of one to six base pairs (bp) that are highly polymorphic. They are abundant in the *P. falciparum* genome, mainly as [TA]n, [T]n, and [TAA]n repeats [75]. Anderson and colleagues pioneered assays of twelve microsatellite loci as sensitive tools for parasite genetic structure and differentiation analyses [76]. These twelve loci have been widely used for assessment of genetic diversity and population structure across many studies. In the context of malaria elimination, microsatellites can be used to fingerprint parasites and resolve relatedness of infections at high spatial resolutions especially in settings with low prevalence and high levels of monoclonality [77–79]. Also, the combination of *P. falciparum* drug-resistance markers and flanking microsatellite loci can be employed to assess the genetic diversity and evolution of selective signatures around drug resistance genes [39].

Similar to *msp/glurp* typing, microsatellite typing depends on accurate DNA fragment amplification by PCR, which can result in artefacts. Accurate allele sizing requires expensive capillary electrophoresis equipment that are largely unavailable. Moreover, there are no methods for phase resolution of parasite haplotypes in mixed infections. However, thousands of *P. falciparum* genome sequences are now available for mining new microsatellite loci which can be developed into new assays for population diversity analyses. Low cost thermocyclers combined with HRM for determining sizes of DNA fragments can improve the availability of microsatellite typing to less specialized laboratories [80].

### Molecular (DNA) barcodes

SNP barcoding has been shown to be robust for evaluating parasite genotypes derived from communities, malaria patients or laboratory strains [81]. It provides a fingerprint for each infection, distinguishing the haplotype signature for single clone infections from those with mixtures of parasite genomes [82]. SNP barcoding are also sensitive for detecting and genotyping sub-microscopic parasitaemia in low malaria transmission areas even when RDTs are negative [83]. In addition, the technique has the potential for identifying the sources of epidemics [84]. The choice of a SNP panel and density within a barcode can be guided by the level of sensitivity required for distinguishing populations or detecting alleles associated with phenotypes such as drug resistance. Early approaches were based on qPCR to distinguish alleles of a panel of 24 SNPs that represented unique signatures for *P. falciparum* isolates [82]. These panels have been developed for qPCR-HRM and the Sequenom Mass Array assays [84, 85]. Only a small number of specialized labs in Africa have successfully implemented and used these assays due to the absence and cost of advanced PCR or array technologies. Several other SNP barcode combinations have now been developed for different types of analyses:

I. A 96 genome-wide SNP panel used to assess the impact of decreased transmission on parasite diversity in Senegal and at the Thai-Burma border [79, 86]. These findings revealed increasingly clonal infections (identical genotypes), due to increased selfing and reduced multilocus recombination as populations declined.
II. A 384-SNP custom GoldenGate Illumina Mass array that clearly resolved global patterns of genetic diversity and the structure of geographically distinct populations across global endemic populations [85].
III. 23-SNP panel for *P. falciparum* containing five mitochondrial and 18 SNPs of the apicoplast genomes thought to offer a higher resolution between isolates from different endemic blocs [87]. The fact that the organelle genome is refractory to recombination makes the mitochondrian/apicoplast SNP panel ideal for mapping the geographic origin of *P. falciparum* isolates. This can identify imported cases of *P. falciparum* into elimination settings.
IV. A recent malaria Taqman Array with 87 loci that enable both species classification and typing of markers across drug resistance and neutral loci [88]. Deployment will be limited by access to the viiA7™ real-time PCR machine. Centres without sequencing and Mass Array facilities can consider this as a viable option for simultaneous drug resistance and diversity typing upon the added benefit of species detection in mixed infections.
V. The Spot malaria project currently provides a large panel of loci including targets for drug resistance and neutral sites for population diversity analysis. This has been constituted into a ‘Genetic Report Card’ (GenRE). This will become increasingly useful as the genotyping methods applied are sensitive enough to detect parasites genomes and alleles in very low-density infections (https://www.malariagen.net/projects/spotmalaria).

While the above techniques and platforms overcome the limitations of traditional *msp/glurp* and microsatellite genotyping, they remain mostly applicable only in specialized northern labs. Furthermore, their sensitivity across populations of different effective sizes and recombination rates, which disrupts association among SNPs panel, remain unevaluated. These techniques are also most suitable for single clone infections. Even with the application of algorithms such as RealMcCOIL to estimate the number of infecting genomes in mixed infections, reconstruction and analysis of individual haplotypes from infections with greater than two clones remain almost impossible [89]. With thousands of *P. falciparum* genomes now available, new barcode panels with population specific alleles combined with new computational models should allow for high resolution determination of origin and diversity of infections in elimination settings and connectivity between spatial transmission hotspots.

### Targeted deep sequencing (TDS)

This is a powerful approach for variant discovery and detection in target genes or genomic regions. TDS can assess the relative distribution of variants of specific genes at individual patient level, or at population level with variance in environment; heterogeneous human and vector hosts as well as interventions. Coupled to long read technologies, it can also permit the reconstruction of haplotypes for relevant genes such as drug resistance loci and vaccine candidates. Applied to known or candidate drug resistance gene loci, it can elucidate the landscape of selection of alleles in such targets as parasites adapt against drugs [90]. In an observational surveillance study of potential drug resistance loci in Senegal and Thailand, TDS identified dozens of previously undescribed mutations in *Pfk13* [91, 92]. TDS can, therefore, be an important public health tool, enabling the detection of low frequency and potential drug resistance loci that may be selected under increased drug pressure.

TDS of *P. falciparum* also provides a high-throughput, highly sensitive approach for detecting minority clones in polyclonal infections and more accurate quantitative estimates of clonal frequency [10, 93]. By determining the frequency of parasite gene haplotypes in individual infections from a parasite population, the tool can enable the assessment of within-host diversity, recombination events and relatedness between infections. Such applications are important in the context of Africa where infections are often mixed, and the ecology of mixed genotypes might influence the transmission of drug resistance loci, the backbone on which resistance may emerge or clones selected by vaccines [94]. However, TDS suffers from ascertainment bias of gene panels. It requires next-generation sequencers, powerful computation and well-trained biostatisticians for data analysis that are not readily available in malaria endemic countries. As this can produce highly relevant data for diversity and drug resistance monitoring in elimination settings, centralized analyses can be done at CEAs and dissemination of simplified reports such as heatmaps of allele and haplotype frequencies could facilitate translation by control programmes.

### Genome-wide variation analysis (GVA)

Global surveys of *P. falciparum* genomic variations has revealed thousands of SNPs, indels, and structural variants, typed across populations using microarray or next-generation sequencing approaches [10, 31]. Genome-wide SNP loci have mostly been characterized and used to scan for and differentiate signatures of natural selection or employed in genome-wide association studies to identify loci underlying phenotypes such as drug resistance [25, 30, 32, 33]. Genome-wide variation typing has evolved from microarray technologies, which suffer from ascertainment bias, to whole genome sequencing (WGS) by short read technologies. Theoretically, the wide variety and density of polymorphisms from WGS should improve the resolution of studies on diversity, the tracking of known functional variants and discovery of novel markers that may be associated with new adaptive parasite phenotypes. WGS data can also be employed in development of new population genetic and diagnostic tools.

Unlike the analysis of individual loci, which may be the target of strong natural selection, inferences from genome-wide diversity are less subject to target biases and, therefore, more accurately reflect overall patterns of *P. falciparum* genetic variation. Nevertheless, the current costs of next-generation sequencers, sample processing reagents, infrastructure for data storage and analysis and need for trained lab and data analyses scientists limits its wide application across most of Africa. In addition, WGS may be limited by the lack of good quality DNA and the need for enrichment for blood samples that contain mostly human DNA. Furthermore, it is difficult to construct haplotypes for complex infections and the technique has a low sensitivity to detect minority clones. With improved error rates from cheaper portable sequencers, such as the nanopore, WGS and GVA will most likely become more translational for malaria elimination. A model where a limited number of CEA laboratories can be equipped to receive and process samples from a network of peripheral laboratories in the continent at high throughput is proposed. Already, WGS is possible in a number of centres in sSA such as the Medical Research Council Unit The Gambia at LSHM. Human capacity for bioinformatics is also being built through African-led networks; Developing Excellence in Leadership and Genetics Training for Malaria Elimination (DELGEME, www.delgeme.org) and the Human Hereditary and disease in Africa (H3Africa) Pan-African Genetic Epidemiology Network (PAMGEN). These programmes will expand access and application of WGS/GVA approaches to malaria elimination research in sSA. Effective deployment and translation could be further facilitated by sample pooling and employing cheaper but sensitive Real time PCR genotyping methods to pre-screen and select samples in peripheral laboratories prior to genome wide analyses at CEAs. Pre-screening could prioritize loci for diversity, drug or vaccine resistance most relevant to the local control and intervention strategies.

## Conclusion

Genetic epidemiology of malaria can improve understanding of parasite origins, flow rates between populations (temporal and spatial), drug resistance and potential susceptibility to candidate anti-malarial drugs and vaccines [26, 29, 95, 96]. Current knowledge on parasite molecular epidemiology has mostly been generated by research with little contribution or guidance from control programmes. This is partly due to limited resources for accessing molecular genotyping technologies and genetic data as a strategy to guide policy, especially in Africa. As it is now clear that malaria parasites deploys their enormous genetic diversity to fight against interventions and host immunity, wider surveys and analyses of parasite genetic variation across different populations will facilitate population specific interventions and provide information to contain resurgence [97]. The tools for generating these genetic data are varied by cost, technological complexity and sensitivity for single nucleotide typing, fingerprinting barcodes, microsatellites, targeted gene variants and whole genome polymorphisms. The choice of the strategy however will depend on the objectives and resources at the disposal of scientists and NMCPs.

Targeted deep and whole genome sequencing are sensitive and specific for detecting patterns of malaria parasite diversity and critical for ‘drug resistant intelligence’. Though they required significant investment in equipment and trained personnel, it is feasible to equip genomic Centres of Excellence in Africa, which will receive and process samples from a network of smaller laboratories with PCR capabilities. These centres will be hubs for developing field deployable genotyping assays for PCR and portable next-Generation sequencing technologies such as the Nanopore to boost genomic surveillance for malaria elimination in Africa. Centres of excellence and molecular surveillance nodes will need computational infrastructure to combine novel approaches for ancestry, relatedness, transmission models and machine learning to resolve spatial–temporal parasite flow, population structure and drug resistance [98, 99]. Thus, genetic epidemiology consortia that integrate with NMCPs, such as the Plasmodium Diversity Network Africa (PDNA) and its training programme for data analysis (DELGEME) represent excellent models for networking and building capacity in sSA for genomics to track and monitor malaria elimination [100].

## Data Availability

Not applicable.

## Abbreviations

sSA: sub-Saharan Africa
CEA: Centres of Excellence in Africa
ACT: artemisinin-based combination therapy
ART-R: resistance to artemisinin derivatives
SEA: South East Asia
PCR: polymerase chain reaction
qPCR: quantitative PCR
HRM: high resolution melting
TWAS: The World Academy of Science
IFS: International Foundation of Science
HRP: histidine-rich protein
RDT: rapid diagnostic test
TES: therapeutic efficacy studies
NMCP: National Malaria Control Programme
WHO: World Health Organization
IC50: inhibitory concentration that kills half of the cultured parasites
WWARN: World Wide Antimalarial Resistance Network
SMC: seasonal malaria chemoprevention
IPTp: intermittent preventive treatment in pregnancy
RFLP: restriction fragment length polymorphism
NGS: next generation sequencing
Gb: gigabyte
AL: artemether–lumefantrine
AS–AQ: artesunate–amodiaquine
MSP: merozoite surface protein
GLURP: glutamine rich protein
COI: complexity of infection
TDS: targeted deep sequencing
GVA: genome-wide variation analysis
WGS: whole genome sequencing
DELGEME: Developing Excellence in Leadership and Genetics Training for Malaria Elimination in sub-Saharan Africa
PAMGEN: Pan-African Genetic Epidemiology Network
PDNA: Plasmodium Diversity Network Africa
AESA: Alliance for Accelerating Excellence in Science in Africa
AAS: African Academy of Sciences
*Pfk13*: *P. falciparum kelch13* propeller gene
*Pfcrt*: *P. falciparum* chloroquine resistance transporter
*Pfmdr1*: *P. falciparum* multidrug resistance protein 1
*Pfmdr2*: *P. falciparum* multidrug resistance protein 2
*Pfubp1*: *P. falciparum* ubiquitin carboxyl-terminal hydrolase 1
Pfap2mu: *P. falciparum* clathrin-associated AP2 adaptor, µ subunit
*Pfdhfr*: *P. falciparum* dihydrofolate reductase
*Pfdhps*: *P. falciparum* dihydropteroate synthetase
